# Marine-Lenhart syndrome combined with papillary thyroid carcinoma: a case report

**DOI:** 10.3389/fendo.2026.1771308

**Published:** 2026-02-23

**Authors:** Yong Zhuang, Wanrong Lin, Qingyan Cai, Huibin Huang

**Affiliations:** 1 Department of Endocrinology, The Second Affiliated Hospital of Fujian Medical University, Quanzhou, China; 2 Department of Endocrinology, Shishi Municipal Hospital, Quanzhou, China

**Keywords:** case report, Grave’s disease, hyperfunctional adenoma, Marine-Lenhart syndrome, papillary thyroid carcinoma

## Abstract

**Background:**

Marine-Lenhart syndrome is a rare clinical condition, and most thyroid nodules combined with Marine-Lenhart syndrome are benign nodules. In this case, Marine-Lenhart syndrome was combined with a malignant nodule and a papillary thyroid carcinoma, which is even rarer.

**Case presentation:** We report a case of Marine-Lenhart syndrome in which the 99m TcO4 scan indicated a “hot nodule” in the thyroid. However, due to the patient’s thyroid ultrasound revealing a TI-RADS 4a categorized nodule, we performed a thyroid fine-needle aspiration biopsy followed by subsequent thyroid lobectomy with isthmusectomy. Both the results of pathological analysis confirmed the presence of papillary thyroid carcinoma.

**Conclusions:**

After confirming the presence of Graves’ disease, it is still essential to consider the possibility of thyroid hyperfunctioning adenoma, namely Marine-Lenhart syndrome. Although most thyroid nodules associated with Marine-Lenhart syndrome are benign and often present as “hot nodules,” it is crucial not to disregard the small probability of thyroid malignancy in such cases.

## Introduction

Graves’ disease is a condition of hyperthyroidism caused by TRAb (1), in which excessive thyroid hormone is synthesized and released by the thyroid gland, resulting in hypermetabolism and sympathetic arousal of the body, causing palpitations, sweating, increased eating and bowel movements, and weight loss. Among patients with Graves’ disease, approximately 10-31% develop thyroid nodules, with the majority of these nodules being non-functioning, while only a small percentage (<5%) are functional nodules (2, 3). Marine-Lenhart syndrome was first reported by Marine and Lenhart as the combination of Graves’ disease with functioning nodules, and it was officially named Marine-Lenhart syndrome by Charkes in 1972 (4).

## Case presentations

A 68-year-old female presented to our department with symptoms of recurrent palpitations, heat intolerance, and excessive sweating without chest tightness, shortness of breath, weight loss, hoarseness, or any other discomfort. Physical examination revealed grade I enlargement of the thyroid gland, with no protrusion of the eyeballs. Stellwag’s sign (-), von Graefe’s sign (-), Joffroy’s sign (-), and Möbius sign (-) were all negative. Hospitalized for thyroid function examination: Thyroid-stimulating hormone (TSH) <0.005 mIU/L (reference range: 0.27-4.2 mIU/L), free triiodothyronine (FT3) 17.9 pmol/L (reference range: 3.1-6.8 pmol/L), free thyroxine (FT4) 56.9 pmol/L (reference range: 12-22 pmol/L), thyroid-stimulating hormone receptor antibody (TRAb) 6.75 IU/L (reference range: <1.75 IU/L), thyroglobulin antibody (TgAb) 15.1 IU/ml (reference range: <115 IU/ml), thyroid peroxidase antibody (TPOAb) 10.7 IU/ml (reference range: <34 IU/ml); electrocardiogram indicates sinus tachycardia. Diagnosis: Graves’ disease. Treatment: Propylthiouracil 50 mg twice daily, propranolol 40 mg once daily.

**Figure 1:**
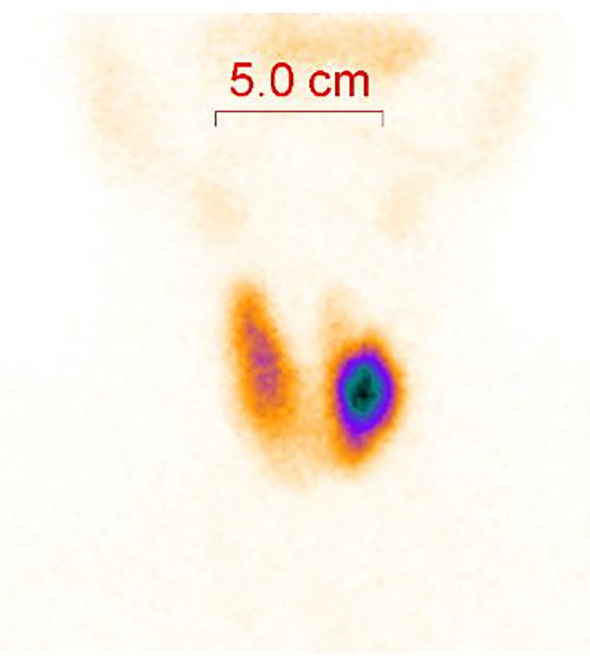
Image from a 99mTcO4 scan.

**Figure 2:**
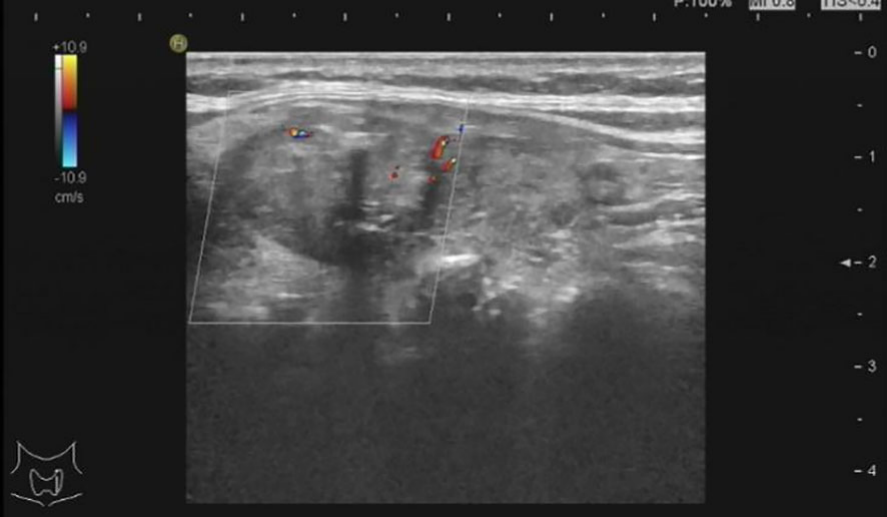
Thyroid color ultrasound image.

During the patient’s stay, a thyroid ultrasound was also performed. The findings are as follows: A heterogeneous hypoechoic lesion is palpable in the upper part of the left thyroid lobe, measuring approximately 1.9 cm × 1.2 cm, with a height-to-width ratio of less than 1. The margins are smooth, and multiple strong echoes with acoustic shadowing, the largest being 0.2 cm, are detected internally. Color Doppler flow imaging (CDFI) shows no blood flow signal within the lesion, but blood flow signals are detectable in the surrounding area (Type II). Conclusion: Solid lesion with multiple internal calcifications in the upper part of the left thyroid lobe (TI-RADS 4a category) (**Figure 1**). Further procedures included a thyroid fine-needle aspiration biopsy and a 99mTcO4 scan. The 99mTcO4 scan findings are as follows: “Thirty minutes after intravenous injection of the radiopharmaceutical, thyroid planar imaging was performed in the anterior position. The thyroid uptake is clear, with a normal position, acceptable size, and clear contours. A nodular radioactive uptake enhancement is observed in the left lobe, with an uptake area of approximately 25.3 × 21.5 mm, and decreased radioactive uptake in the upper pole. The radioactive uptake in the right lobe is still acceptable. Conclusion: ‘Hot nodule’ in the left lobe of the thyroid, suggestive of a hyperfunctional adenoma.” (**Figure 2**). Therefore, the diagnosis is corrected to Marine-Lenhart syndrome.

**Figure 3:**
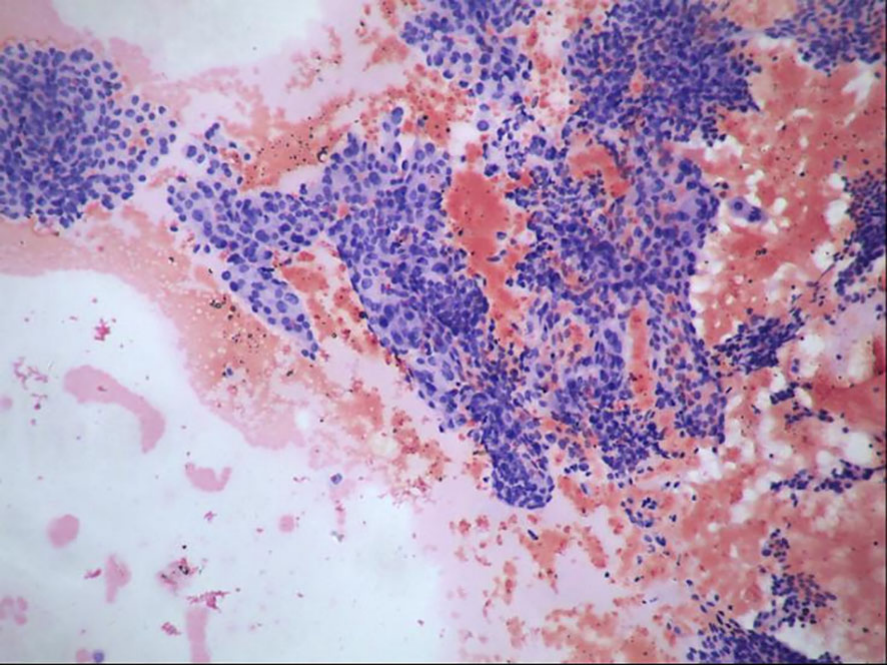
Pathological image of thyroid nodule fine-needle aspiration biopsy.

**Figure 4:**
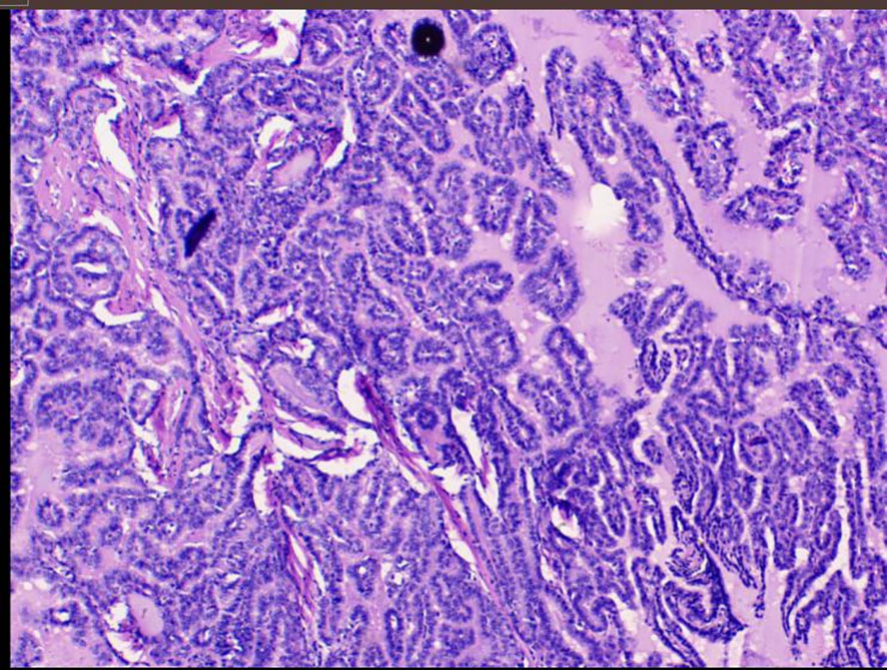
Pathological image of resected thyroid nodule.

Subsequently, the pathology report of the fine-needle aspiration biopsy of the left thyroid nodule revealed the following: The smear shows a scattered and clustered arrangement of thyroid follicular epithelial cells. The cell arrangement appears crowded and occasional nuclear grooves and intranuclear inclusions are observed. Papillary thyroid carcinoma cannot be ruled out (TBS classification: Category V) (**Figure 3**). The patient agreed to undergo surgical resection for treatment and continued to receive “50mg of propylthiouracil twice daily (bid) and 40mg of propranolol once daily (qd)” as preparation before the operation. After three weeks of treatment, a follow-up thyroid function examination showed FT4 at 16.88 pmol/L, FT3 at 7.65 pmol/L, and TSH at 0.005 mIU/L. Consequently, the patient underwent “left thyroid lobectomy with isthmusectomy plus radical neck lymph node dissection” by the thyroid surgery department. After the surgery, the patient no longer took “propylthiouracil” and was given “50 ug of levothyroxine once daily (qd)” as replacement therapy. Pathology report of the surgical resection: The thyroid nodule was diagnosed as papillary thyroid carcinoma with focal calcification. It measures approximately 2cm in diameter and infiltrates the surrounding fibrofatty and striated muscle tissues. It involves blood vessels and adjacent parathyroid tissues but does not show definite neural invasion. Background nodular goiter changes are present. Regarding the lymph nodes in the left central neck area, two lymph nodes were examined, and no cancer metastasis was found (**Figure 4**).

The patient had a smooth postoperative recovery without any complications and was discharged successfully. Follow-up visits were conducted at our department on the 7th and 14th days after discharge. The patient continued to receive treatment with “50 ug of levothyroxine sodium tablets once daily (qd)”. The patient did not report any complaints, such as palpitations, heat intolerance, or excessive sweating. On the 14th day post-surgery, thyroid function tests showed FT4 at 15.77 pmol/L, FT3 at 5.64 pmol/L, and TSH at 0.61 mIU/L.

## Discussion

When a “hot nodule” appears on thyroid scintigraphy with elevated TSH, it is a typical presentation of Marine-Lenhart syndrome, showing TSH dependence. On the other hand, atypical Marine-Lenhart syndrome refers to thyroid nodules that are non-TSH dependent, with autonomous nodule function. In the case of low TSH levels, the thyroid nodules still exhibit functionality, and scintigraphy indicates a “hot nodule” (5–7). The imaging manifestations of atypical cases may include: 1) heterogeneous uptake in the diffusely hyperactive area or poorly defined focal concentration; 2) under the background of diffusely increased uptake, the autonomous functioning thyroid nodule(s) may appear as “warm” or “cold” nodules due to radioiodine uptake competition; 3) serum TRAb levels below the diagnostic threshold for typical Graves’ disease. In this case of Graves’ disease patient, thyroid scintigraphy showed “hot nodules” when TSH was decreased, which fell under the category of atypical Marine-Lenhart syndrome.

The pathological results of thyroid nodules in Marine-Lenhart syndrome are generally benign (8). Additionally, most thyroid cancers appear as “cold nodules” on radionuclide scans, while the occurrence of “hot nodules” is rare, approximately 4.7% to 8.2% (9). However, in the case of Marine-Lenhart syndrome, although the patient’s thyroid nodules indicated “hot nodules,” they were malignant tumors. Furthermore, studies have shown that highly functional thyroid malignancies are often follicular carcinomas and are frequently accompanied by metastasis (10). In contrast, the pathological findings in this case indicated papillary thyroid carcinoma without metastasis, which was rare.

The treatment options for Marine-Lenhart syndrome include antithyroid medication, radioactive iodine (131I) therapy, and surgical treatment. In this patient’s case, the thyroid fine-needle aspiration biopsy suggested a high possibility of papillary thyroid carcinoma. Therefore, surgical treatment is appropriate.

## Conclusion

Considering the possibility of Marine-Lenhart syndrome in patients with Graves’ disease is crucial. While most thyroid nodules associated with Marine-Lenhart syndrome are benign and typically present as “hot nodules,” it is essential not to overlook the possibility of thyroid cancer.

## Data Availability

The original contributions presented in the study are included in the article/supplementary material. Further inquiries can be directed to the corresponding author.
